# Bladder cancer biomarkers: Past and future directions

**DOI:** 10.1002/bco2.61

**Published:** 2021-01-04

**Authors:** Miguel Rodriguez‐Homs, Janet Baack Kukreja

**Affiliations:** ^1^ Division of Urology University of Colorado Aurora CO USA

Novel biomarkers to detect malignancy have become increasingly studied to evaluate their use in clinical practice. Within bladder cancer, several biomarkers have been investigated and become available for use in initial patient work‐up, treatment responses and algorithms, and surveillance. However, current AUA and EAU guidelines caution against the use of biomarkers in place of cystoscopy for bladder cancer surveillance. Their roles in practice include consideration in assessing a response to BCG and equivocal cytology.

Recently, Vasdev et al submitted a manuscript entitled “The role of URO17^TM^ biomarker to enhance diagnosis of urothelial cancer in new hematuria patients—First European Data” to which this editorial will pertain. In this study, the authors investigated the use of K17 (Keratin 17), an oncoprotein expressed in several malignancies, as a marker for early detection of bladder cancer in gross hematuria patients without prior urologic oncology history. Prior studies have shown promise of its use as a marker as it is expressed in greater than normal quantities in urothelial carcinoma compared to normal bladder mucosa.^1^

The study evaluated 71 patients in a prospective blinded fashion in which a first morning void urine sample in addition to patient demographics were obtained prior to standard of care cystoscopy with bladder biopsy in the operating room for gross hematuria workup. Samples were prepared and stained using URO17^TM^ for 20 minutes. Results demonstrated a sensitivity 100%, specificity 92.6%, positive predictive value (PPV) 0.957, and negative predictive value (NPV) 1.

While this study shows promise with URO17^TM^, multiple biomarkers have shown early promise and later have not been able to be implemented on a wide scale. Although the results here are very impressive, the authors had 71 symptomatic patients in their cohort, this sample size might be too small to draw concrete conclusions for implementation in practice. They also report that a NPV of 1 offers potential for a screening tool in the primary care setting to potentially reduce unnecessary urologic referrals and invasive procedures. While a negative result may suggest a low likelihood of malignancy, there are other urologic causes of gross hematuria that warrant evaluation. These include urolithiasis, vascular anomalies, prostate etiology, unrecognized trauma, and so on. There is an approximately 14% and 3% incidence of urinary tract malignancy in patients with gross hematuria and microscopic hematuria, respectively.^2^ Gross hematuria should almost always prompt a referral to urology.

An aspect important to discuss is the histology of urologic cancers detected in this cohort. All 44 patients with malignancy in this study were of urothelial histology albeit of varying stages and grades. In addition, Babu et al included only classic urothelial carcinoma in their study demonstrating the possible nature of K17 as a biomarker for bladder cancer.^1^ Urothelial carcinomas comprise about 90% of bladder cancer histology, which leaves approximately 10% of bladder cancers with nonurothelial origin. Additionally, up to 25% of patients are diagnosed with variant histology.^3^ The proposed URO17^TM^ biomarker has not been studied or shown efficacy in the nonurothelial histology population. Given the data, there is a sizable group of patients at risk of delayed or missed diagnosis if biomarker screening was implemented without imaging and cystoscopy. However, as an adjunct to cystoscopy, URO17^TM^ seems extremely promising and the results of validation will be anxiously awaited.

Currently, there is insufficient evidence to support screening for bladder cancer in asymptomatic patients. Population based screening needs to have a high enough prevalence of disease and early detection results in lower morbidity and mortality. Although this may be applicable in colorectal cancer, this has yet to be demonstrated in bladder cancer. The majority of bladder cancers diagnosed are superficial and may never progress to muscle‐invasive disease which would make them not ideal for population based screening.^4^ Thus, a negative biomarker screening test in an asymptomatic patient would not alter management while a negative result in a symptomatic patient would still likely lead to further work‐up including cystoscopy. Previous bladder cancer biomarkers have not been implemented in clinical practice because there is little alteration in the outcomes and they often increase the costs of work‐up.

That brings us to the question, what is the future role of URO17^TM^ in clinical practice? There is much promise with using a highly sensitive and highly specific test with surveillance in patients with known bladder cancer monitoring for recurrence. Cytology is currently widely used as an adjunct with other diagnostic tests given its low sensitivity for low‐grade urothelial carcinoma. Though relatively inexpensive, cytology provides assistance in identifying high‐grade recurrence in the urinary tract with high specificity.^5^ A role for URO17^TM^ in this setting could prove to be very useful. Furthermore, care must be taken to not use it in place of cystoscopic evaluation. While at times inconvenient for the patient, cystoscopy is the gold standard for evaluating bladder pathology and more so in the setting of advancements in detection (ie, blue light). A false negative biomarker test without cystoscopy could delay diagnosis and allow progression of bladder cancer.

Overall, the results of Vasdev's et al's study are quite impressive with promising results. One must be cautious in applying this preliminary data to clinical practice for reasons stated above. Despite these concerns, this study along with others demonstrate the advances in inquiries within the field of bladder cancer with the potential to shape management and outcomes.

## CONFLICT OF INTEREST

None.

## References

[1] Babu S , Mockler DC , Roa‐Peña L . Keratin 17 is a sensitive and specific biomarker of urothelial neoplasia. Mod Pathol. 2019;32:717–24. 30443013 10.1038/s41379-018-0177-5

[2] Tan WS , Feber A , Sarpong R , Khetrapal P , Rodney S , Jalil R , et al. Who should be investigated for haematuria? Results of a contemporary prospective observational study of 3556 patients. Eur Urol. 2018;74(10):10–14. 29653885 10.1016/j.eururo.2018.03.008

[3] Klaile Y , Schlack K , Boegemann M , Steinestel J , Schrader AJ , Krabbe LM . Variant histology in bladder cancer: how it should change the management in non‐muscle invasive and muscle invasive disease? Transl Androl Urol. 2016;5(5):692–701. 27785426 10.21037/tau.2016.06.13 PMC5071184

[4] U.S. Preventative Services Task Force . Screening for bladder cancer: recommendation statement. Am Fam Physician. 2012;85(4):397–9. 22335320

[5] Xing J , Reynolds JP . Diagnostic advances in urine cytology. Surg Pathol Clin. 2018;11(3):601–10. 30190143 10.1016/j.path.2018.06.001

